# Health policy challenges in Lebanon’s healthcare system: on sexual and reproductive health and rights

**DOI:** 10.1080/26410397.2025.2525600

**Published:** 2025-07-02

**Authors:** Faysal El Kak, Sohayla El Fakahany, Tamar Kabakian-Khasholian, Stephen McCall, Ghada Saad

**Affiliations:** a Senior Lecturer, Health Behavior and Sexuality & Public Health, Faculty of Health Sciences, The American University of Beirut, Beirut, Lebanon; b Social Research Consultant, Faculty of Health Sciences, The American University of Beirut, Beirut, Lebanon.; c Associate Professor, Department of Health Promotion and Community Health, The Faculty of Health Sciences, The American University of Beirut, Beirut, Lebanon; d Instructor of Public Health Practice, The Center for Research on Population and Health, The American University of Beirut, Beirut, Lebanon; e Director, The Center for Research on Population and Health, The American University of Beirut, Beirut, Lebanon

**Keywords:** sexual and reproductive health and rights (SRHR), Lebanon healthcare system, health strategy and policy, family planning, comprehensive sexuality education (CSE), gender equity in healthcare

## Abstract

Lebanon’s healthcare system has demonstrated remarkable resilience amidst ongoing political and economic turbulence. Yet, the critical domain of Sexual and Reproductive Health and Rights (SRHR) remains underserved. This commentary analyses the systemic barriers, policy deficiencies, and urgent needs that shape SRHR within Lebanon's healthcare landscape. Despite the country’s commitments to international frameworks like the ICPD and CEDAW, SRHR policies are hindered by political fragmentation, societal conservatism, and insufficient prioritisation. These challenges translate into inadequate and inconsistent family planning services, a lack of comprehensive sexuality education, inadequate maternal healthcare, and significant obstacles in accessing essential services, especially for marginalised communities such as refugees, women, and youth. Lebanon’s “Vision 2030” health strategy, while ambitious in scope, offers only limited engagement with SRHR, which leaves systemic inequities unaddressed. Renewed episodes of violence and displacement further strain the healthcare system and deepen the disparities faced by vulnerable groups. The reliance on temporary, NGO-led initiatives to fill gaps in service provision underscores a broader policy paralysis and inconsistent resource allocation, which together prevent the sustainable integration of SRHR into national health frameworks. This commentary calls for a gender-sensitive, inclusive healthcare policy that positions SRHR as a foundational pillar of public health, gender justice, and social equity. Achieving this requires concerted efforts among government agencies, NGOs, and international partners to overhaul existing frameworks and address structural barriers.

## Résumé

Le système de santé libanais fait preuve d’une remarquable résilience face aux turbulences politiques et économiques actuelles. Pourtant, le domaine crucial de la santé et des droits sexuels et reproductifs reste mal desservi. Ce commentaire analyse les obstacles systémiques, les carences des politiques et les besoins urgents qui façonnent la santé et les droits sexuels et reproductifs dans le paysage sanitaire libanais. Malgré les engagements du pays envers des instruments internationaux comme le Programme d’action de la CIPD et la Convention sur l’élimination de toutes les formes de discrimination à l’égard des femmes, les politiques de santé et de droits sexuels et reproductifs sont entravées par la fragmentation politique, le conservatisme sociétal et une priorisation insuffisante. Ces défis se traduisent par des services de planification familiale inadéquats et incohérents, un manque d’éducation complète à la sexualité, des soins de santé maternelle inadaptés et d’importants obstacles à l’accès aux services essentiels, en particulier pour les communautés marginalisées telles que les réfugiés, les femmes et les jeunes. La stratégie de santé du Liban, « Vision 2030 », bien qu’ambitieuse, n’offre qu’un engagement limité en matière de santé et de droits sexuels et reproductifs, ce qui ne permet pas de traiter les inégalités systémiques. La recrudescence des épisodes de violence et des déplacements de population met à rude épreuve le système de santé et creuse les disparités auxquelles sont confrontés les groupes vulnérables. Le recours à des initiatives temporaires dirigées par des ONG pour combler les lacunes dans la prestation de services met en évidence une paralysie politique plus large et une allocation incohérente des ressources, ce qui empêche l’intégration durable de la santé et des droits sexuels et reproductifs dans les cadres nationaux de santé. Ce commentaire recommande une politique de santé inclusive et sensible au genre qui positionne la santé et les droits sexuels et reproductifs comme un pilier fondamental de la santé publique, de la justice de genre et de l’équité sociale. Pour y parvenir, des efforts concertés entre les institutions gouvernementales, les ONG et les partenaires internationaux sont nécessaires en vue de remanier les cadres existants et lever les barrières structurelles.

## Resumen

El sistema de salud del Líbano ha demostrado notable resiliencia en medio de una continua turbulencia política y económica. Sin embargo, el dominio crítico de Salud y Derechos Sexuales y Reproductivos (SDSR) continúa desatendido. Este comentario analiza las barreras sistémicas, las deficiencias normativas y las necesidades urgentes que definen SDSR dentro del panorama sanitario del Líbano. A pesar de los compromisos del país con marcos internacionales como la CIPD y CEDAW, las políticas de SDSR son obstaculizadas por la fragmentación política, el conservadurismo social e insuficiente priorización. Estos retos se traducen en servicios de planificación familiar inadecuados y desiguales, una falta de educación integral en sexualidad, servicios de salud materna inadecuados y considerables obstáculos para acceder a los servicios esenciales, especialmente para las comunidades marginadas tales como refugiados, mujeres y jóvenes. La estrategia sanitaria del Líbano, “Visión 2030”, aunque ambiciosa en su alcance, ofrece solo un compromiso limitado con SDSR, que deja las inequidades sistémicas sin abordar. Episodios renovados de violencia y desplazamiento suponen una carga mayor para el sistema de salud y aumentan las disparidades enfrentadas por grupos vulnerables. La dependencia de iniciativas temporales lideradas por ONG para subsanar las deficiencias en la prestación de servicios subraya una parálisis normativa más amplia y una asignación de recursos incongruente, que juntas impiden la integración sostenible de SDSR en los marcos sanitarios nacionales. Este comentario exige la formulación de una política sanitaria más inclusiva y sensible al género, que posicione SDSR como el pilar fundacional de la salud pública, la justicia de género y la equidad social. Para lograrlo, se necesitan esfuerzos concertados entre agencias gubernamentales, ONG y socios internacionales para reformar los marcos existentes y abordar las barreras estructurales.

## Introduction

Amid ongoing economic and political turmoil, Lebanon's healthcare system has exhibited remarkable strength and flexibility; this can be attributed to continuous efforts of the Ministry of Public Health (MoPH) to strengthen the primary health care system, and assistance from international and local non-governmental organisations (NGOs).^1^ This resilience ensures the availability of critical services, such as emergency care, vaccination programmes, and maternal and child health services, even in times of crisis.^2,3^ Nevertheless, gaps remain in the provision of sexual and reproductive health and rights (SRHR) mainly due to the healthcare systems’ inability to move towards international commitments and national policies as reflected in Lebanon’s commitments to frameworks such as the International Conference on Population and Development (ICPD) and the Convention on the Elimination of All Forms of Discrimination Against Women (CEDAW). Despite these commitments, these frameworks are inadequately integrated into national policies due to internal resistance that stems from conservative religious and traditional institutions and limited political prioritisation.^4,5^ The Universal Periodic Review (UPR) on Lebanon’s Sexual and Reproductive Rights highlights several legal and policy gaps that undermine SRHR protections, including reservations on Article 16 of CEDAW, which limits gender equality in family law, and refusing to ratify key treaties such as the 1951 Refugee Convention and the Convention on the Rights of Persons with Disabilities (CRPD). National laws also continue to criminalise homosexuality (Article 534), restrict access to abortion (Articles 539–549), and fail to criminalise marital rape and sexual harassment. Additionally, Lebanon has no civil code. The country’s legal system relies on 15 separate personal religious-based status laws and courts.^6^ These sectarian personal status laws result in inequality between Lebanese citizens and further entrench gender inequalities, particularly in marriage, divorce, inheritance, and custody matters, as the personal status laws of all religious confessions place and treat women as inferior to men.^7^

This gap in SRHR policy results in a myriad of issues, including but not limited to, lack of access to family planning, the absence of comprehensive sexuality education, increased maternal mortality rates, stagnated contraceptive prevalence, unsafe terminations of pregnancy, non-standardised post-abortion care, and inadequate screening and prevention of sexually transmitted infections (STIs).^8,9^ Furthermore, these challenges are exacerbated by resistance from religious and conservative institutions, whose narratives and discourses shape national policies. The insufficient political will to prioritise SRHR continues to hinder progress. Instead, Lebanon relied historically on operational guidelines and most recently broad strategic frameworks, such as the *Vision 2030 Health Strategy*, which prioritise general public health goals but fail to address the nuanced needs of its diverse population. This fragmented approach to SRHR policy development positions the country far from aligning with its global commitments on rights, including sustainable development goals (SDGs) SDG3, targeting good health and well-being, and SDG5, on gender equality and empowerment of all women and girls. It also creates a challenging healthcare environment for marginalised populations, who often face barriers to accessing comprehensive reproductive and sexual healthcare.^7^ This commentary aims to analyse the systemic barriers, policy deficiencies, and urgent needs that shape SRHR within Lebanon's healthcare landscape, with a focus on the Lebanon Healthcare Vision 2030. It also calls for a gender-sensitive, inclusive healthcare policy that positions SRHR as a foundational pillar of public health, gender justice, and social equity.

## Political and contextual background on SRHR in Lebanon

Lebanon’s political system is structured around a confessional power-sharing arrangement, which fragments policymaking across religious and political lines, and thereby complicates the development of formal and unified healthcare policies. The Ministry of Public Health (MoPH) is the primary healthcare authority, but political, sectarian, religious, and conservative influences constrain its directives.^10^ SRHR, in particular, faces significant challenges due to a lack of cohesion across government ministries. The Ministry of Education and Higher Education, Ministry of Justice, and Ministry of Labor have not incorporated SRHR into their policies or in school curricula, which results in a broader challenge of insufficient acknowledgment of SRHR within state planning.^7^ Adding to this are the outdated laws related to personal status and SRHR, including laws governing child marriage, age of consent, marital rape, abortion, and forced marriage, which remain in urgent need for reform.^11^ The confessional political system enables religious institutions to block legislative progress and prevent reforms that would align Lebanon’s legal framework with international human rights standards. What is often lacking in policy development is, at times, addressed through programmes and service provisions that are often offered by NGOs and sometimes supported by MoPH. These initiatives step in to fill the gaps left by incomplete or underdeveloped policies and offer practical solutions and immediate support to underserved populations. However, such initiatives remain fragmented and vulnerable to political shifts, as they are often subject to sectarian influences and donor-driven funding priorities rather than sustained governmental commitment.^10^

Lebanon’s healthcare system remains focused on fragmented measures rather than comprehensive healthcare delivery, which leads to unclear roles and responsibilities within the healthcare continuum.^12^ The available programmes and services that are implemented with an ongoing lack of policy involve a number of stakeholders, including government ministries, international organisations, and local NGOs. These include the United Nations Population Fund (UNFPA), and the World Health Organization that collaborate with the MoPH to advance SRHR through family planning, STI screening, prenatal care, and mental health initiatives. Some progress has been made through such collaborations, but they are often largely limited to specific projects or transient programmes. Organisations such as PLAN International aim to improve education for girls and young women and the International Development Research Centre (IDRC) offers research grants for marginalised groups. Nonetheless, the confessional arrangement in Lebanon, cross-sectarian political fragmentation, conservative discourses, and inadequate citizen engagement in the decision-making process directly result in policy paralysis and deadlock.^13^ Due to this predicament where SRHR issues lack the fortitude and backing of political endorsement that is necessary for a sustained effort, marginalised population groups such as youth, women and refugees usually face consequential effects and are forced to depend on external aid that is often limited to specific programmes, rather than their healthcare and rights being fully embedded in national policy frameworks.

## Blurry vision 2030: analysis of Lebanon’s SRHR policy gaps

Against the complex socio-political backdrop, the MoPH, with support from the WHO, developed the *National Health Strategy: Vision 2030* to promote universal health coverage and health security. Lebanon’s Vision 2030 Health Strategy was introduced as a long-term roadmap for the healthcare sector, and it largely aims to enhance health governance, service delivery, and financial sustainability. The strategy is divided into strategic directions, strategic goals, strategic objectives, and strategic investments. While the strategy emphasises universal health coverage, digital transformation, and workforce capacity-building in five strategic directions, 24 goals, and 82 objectives, it largely overlooks critical SRHR components such as family planning, access to safe abortion, and comprehensive sexuality education.^14^

A review conducted one year after the launch of the 2030 health strategy reveals significant shortcomings in its implementation. Out of 88 identified objectives, only one has been fully completed.^2^ This could be due to continuous political instability and limited resources. Furthermore, it also highlights the challenges in operationalising the strategy's goals and suggests a need for more focused and realistic planning. The strategy also neglects the health needs of children (beyond basic immunisations), adolescents (with a glaring absence of youth-friendly services), older adults, persons with disabilities, and women (apart from efforts to reduce maternal mortality and provide some basic reproductive health services, with no clear objective on how to improve women’s or reproductive health). In fact, there is no clear life course approach which is being adopted globally. Overall, the strategy falls short of directly addressing SRHR. Strategic objective 4.1.3 is the only objective referencing SRHR and it aims to address social health determinants and equity, restore progress in SDG3 (focusing on child and maternal health), and include vulnerable groups such as older adults, persons with disabilities, prisoners, and refugees, with particular attention to gender equality.^2^ This involves a focus on vulnerable groups, and a superficial mention of gender equality. Several projects and interventions targeting vulnerable populations have been implemented, such as programmes to prevent financial hardship through hospitalisation coverage for lifesaving and limb-saving conditions, as well as neonatal intensive care. The other section that includes SRHR is the strategic investment area 1.2, which focuses on healthier living and disease prevention and claims to monitor and promote population health through priority programmes targeting non-communicable diseases, maternal, adolescent, and child health, as well as nutrition. However, this is ostensibly to be achieved by setting up registries and monitoring systems via partnerships, a goal that has shown no tangible results, as no progress in this area has been reported in the 1-year review.^2^ The strategy also did not echo or cover the SRHR links that have been made within SDGs 3 and 5.

The Vision 2030 Health Strategy’s lack of focus on SRHR is a major oversight. Instead of a holistic approach to women’s health, it narrowly addresses maternal health and omits critical areas like family planning, contraception, sexual health, STI prevention, and safe abortion care. This limited scope fails to address essential aspects of women’s health, highlighting the challenge of translating strategy into action amidst political instability and limited resources. The strategy’s omissions are evident in its disregard for family planning and contraception and largely sidelining women outside of maternal care contexts. The gap between policy formulation and execution, worsened by resource constraints and political instability, often results in ineffective strategies.^15^

## Analysis of SRHR needs and policy implications

Family planning has remained one of the most overlooked and the most poorly understood SRHR requirements in Lebanon. Family planning frameworks do not feature a clearly defined ideology in the government’s policies; it has become a mere collection of services instead of a positive fundamental for women’s reproductive health and well-being. One thing that can partly explain the limited national focus on family planning in Lebanon is the decrease in the Total Fertility Rate (TFR), which has declined to nearly replacement level.^16^ Despite this overall decline in TFR, pockets of high fertility persist across the country, which shows the continued need for equitable access to family planning services and education to address disparities and support reproductive rights. Family planning is not merely about reducing fertility rates and offering contraception services, it is concerned with improving women’s agency and autonomy, reducing maternal and child mortality, and contributing to sustainable population growth. However, without such clear understanding of family planning as an ideology that supports informed choice and individual rights, Lebanon’s approach fails to prioritise these principles.^9,17,18^ This results in access to family planning services being inconsistent, with disparities between urban and rural areas. In many regions, especially those with high refugee populations, SRHR services remain fragmented and inadequately funded.^19^ This lack of a cohesive family planning ideology not only limits contraceptive options but also reflects a broader resistance to framing reproductive rights within a rights-based and inclusive framework. It is not merely a gap in service provision but a significant oversight in acknowledging family planning as a critical component of social empowerment and gender equality.

Another critical SRHR issue is the absence of comprehensive sexuality education (CSE). CSE is crucial for equipping young people with knowledge and skills to make informed decisions about SRHR, and its exclusion further highlights Lebanon’s inadequate approach to SRHR policy.^13^ The Lebanese Ministry of Education and Higher Education (MEHE) in collaboration with the MoPH and UNFPA had a school-based reproductive health and gender curriculum developed which was then adopted by the government in 2007 towards passing Decree 6610/11 in 2010.^20^ Topics included the menstrual cycle, reproductive anatomy and physiology, STIs, contraception and reproductive rights. During this time, similar and consistent efforts have been made to normalise CSE for Lebanese youth and citizens broadly.^21,22^ Although the curriculum and other CSE initiatives were rooted in scientific principles and approached cultural sensitivities carefully, they faced opposition from religious and conservative leaders, which prevented the adoption of CSE in most schools and ultimately resulted in removal.^20^ Currently, the only SRHR-related subject taught in schools is “The Human Body and Development,” forcing students to seek information on topics like gender, reproductive health, violence prevention, bodily autonomy, and sexuality outside formal education.^23^ This absence of CSE perpetuates misinformation, leaves youth without essential guidance on sexual health, and contributes to increased rates of unintended pregnancies, sexually transmitted infections (STIs), and gender-based violence (GBV), all of which have lasting impacts on mental health.^7^

Refugee populations, particularly Syrians and Palestinians, add complexity to Lebanon’s SRHR landscape.^19^ Although basic healthcare needs are partially covered through UNHCR and UNRWA supported programmes, refugees encounter considerable cultural and financial barriers that limit their access to adequate SRHR services.^24,25^ Lebanon’s restrictive refugee policies also exacerbate these barriers, with measures like the 2014 Policy Paper on Syrian Refugee Displacement and the 2015 suspension of new UNHCR registrations creating significant healthcare access challenges.^26^ Without comprehensive health policies that address the unique needs of refugee populations, vulnerable women face increased risks of GBV, unintended pregnancies, and unsafe deliveries.^27–30^ Ultimately, without an ideological commitment to SRHR, Lebanon’s healthcare system remains reactive rather than transformative as it misses a critical opportunity to integrate SRHR as a core principle and foundational element of public health and social equity.

## Impact of renewed violence on SRHR in Lebanon

To compound the above-described challenges and SRHR needs, Lebanon has been enduring hostilities, a war, and continuous airstrikes by Israel for over a year. This has further strained an already limited healthcare landscape and disproportionately impacted women. Lebanon witnessed significant displacement, with over 1.2 million people displaced nationwide.^31,32^ As of 30 September 2024, the Displacement Tracking Matrix (DTM) identified 346,209 IDPs – with over 31,000 families (∼137,000 individuals) living in 820 collective shelters.^33^ More than half (52%) of all internally displaced people in Lebanon are women and girls. This includes 13,900 pregnant women who have been impacted by the attacks in Lebanon, around 1,550 of whom are due to deliver over the next few months. This comes as around a quarter of the country's infrastructure has been destroyed.^34^ The healthcare system, already strained before the crisis, is now at a critical breaking point with around 100 primary healthcare centres and dispensaries shut down, along with several hospitals.^34^ These conditions exacerbate existing vulnerabilities, particularly concerning SRHR. Displaced women encounter significant barriers in accessing hygienic and sanitary products, antenatal care, safe childbirth, and postpartum support, which heightens risks for SRHR and maternal complications, including infections and preterm births.^35,36^ The crisis also poses severe challenges to contraception access, as displaced women, especially those in informal shelters, experience limited availability of resources despite the MoPH's efforts to maintain supply chains.^37^ Also, the stigma surrounding contraception use in these settings further hinders effective SRH service delivery, which puts displaced women at risk of unintended pregnancies.^38^ Additionally, the escalation of GBV is alarming, with displaced women and girls being particularly susceptible to sexual violence, exploitation, and abuse in overcrowded shelters where they share small spaces with strangers and have no privacy.^39,40^ Mental health support is another critical challenge for displaced women, who often suffer from heightened anxiety, depression, fear, loss, and trauma. These issues are frequently aggravated by family separation, evictions from homes, and economic hardships.^41^ To address this, the MoPH, in collaboration with UNHCR and UNFPA, has established mental health focal points in certain shelters to identify and refer individuals in need of care.^42^ Additionally, rape survivors are being directed to specialised centres for comprehensive management and support.^43^ While international organisations, local NGOs, and the MoPH have mobilised initiatives such as mobile health units, emergency obstetric care, and safe spaces for survivors of GBV, these services fall far short of meeting the urgent needs of the displaced population.

## Conclusion

Lebanon faces substantial challenges in developing effective policies, but addressing specific healthcare issues is essential for improving equity outcomes across its population. The absence of formal policies impedes Lebanon's ability to meet its international commitments. The Vision 2030 Health Strategy, while useful as a broad framework, has proven insufficient in addressing complex needs. To move beyond the limitations of broad agendas such as Vision 2030, Lebanon requires a comprehensive healthcare policy that highlights and directly addresses SRHR in a framework that is gender-sensitive, inclusive, actionable, and capable of addressing the needs of all citizens. By focusing on policy innovation, resource mobilisation, and capacity building, Lebanon can advance SRHR and improve healthcare outcomes across all demographics. This effort will require collaboration among government agencies, NGOs, and international organisations. Such a policy could transform Lebanon’s healthcare landscape by creating a structure that not only meets immediate SRHR needs but also adapts to the shifting dynamics of conflict and displacement as Lebanon continues to face uncertainty. Strengthening reproductive health services, ensuring GBV protections, and expanding mental health support in conflict-prone regions also stand as critical steps to safeguard the well-being and dignity of women during this turbulent period.

## Positionality

The authors of this paper come from different academic and practice-based backgrounds, spanning public health, anthropology, and epidemiology. Collectively, the team shares a sustained commitment to advancing sexual and reproductive health and rights (SRHR) in the Arab region, particularly in Lebanon. We recognise that our positions within academic institutions, and our affiliations with SRHR advocacy in the region, have shaped the questions we posed and the interpretive lenses we applied throughout this study.

## Provenance statement

This article was not commissioned and went through external peer review.
